# Supplementary material for: An open-source framework for large-scale, flexible evaluation of biomedical text mining systems
Source: J Biomed Discov Collab. 2008 Jan 29;3:1. doi: 10.1186/1747-5333-3-1 (PMC2276192; doi:10.1186/1747-5333-3-1)
Supplement: Additional file 1 — Evaluation platform source code. The additional file contains the software evaluation framework discussed in this paper. The most current release can be downloaded from SourceForge.net [1]. See the accompanying README file for instructions on installation and use. Also included in the distribution is a collection of UIMA wrappers for some commonly used BioNLP tools and annotated corpora. [file 1747-5333-3-1-S1.zip › bionlp_uima_repository-1.0.1/docs/api/src-html/edu/uchsc/ccp/util/nlp/mention/comparison/ClassMentionComparator.html]

```
001    package edu.uchsc.ccp.util.nlp.mention.comparison;
002    
003    import edu.uchsc.ccp.util.nlp.annotation.comparison.SpanComparator;
004    import edu.uchsc.ccp.util.nlp.mention.ClassMention;
005    
006    public interface ClassMentionComparator {
007    
008        /**
009         * 
010         * @param classMention1
011         * @param classMention2
012         * @param maximumComparisonDepth
013         * @param depth
014         * @param spanComparator
015         * @param compareLinkedAnnotationSpans flag allowing spanless comparison 
016         * @return
017         */
018        public int compare(ClassMention classMention1, ClassMention classMention2, SpanComparator spanComparator, int maximumComparisonDepth);
019    }
```
